# The ERG1a potassium channel increases basal intracellular calcium concentration and calpain activity in skeletal muscle cells

**DOI:** 10.1186/s13395-019-0220-3

**Published:** 2020-01-16

**Authors:** Clayton Whitmore, Evan P.S. Pratt, Luke Anderson, Kevin Bradley, Sawyer M. Latour, Mariam N. Hashmi, Albert K. Urazaev, Rod Weilbaecher, Judith K. Davie, Wen-Horng Wang, Gregory H. Hockerman, Amber L. Pond

**Affiliations:** 1 Anatomy Department, Southern Illinois University School of Medicine, Carbondale, IL 62902 USA; 2 Medicinal Chemistry and Molecular Pharmacology, Purdue University, West Lafayette, IN 47906 USA; 3 Doisey College of Health Sciences, Saint Louis University, St. Louis, MO 63103 USA; 4 School of Liberal Arts, Sciences and Education, Ivy Tech State college, Lafayette, IN 47905 USA; 5 Biochemistry Department, Southern Illinois University School of Medicine, Carbondale, IL 62902 USA; 6 Gene Editing Core Facility, Purdue University, West Lafayette, IN 47906 USA; 7 Southern Illinois University, 1135 Lincoln Drive, Carbondale, IL 62902 USA

**Keywords:** ERG1A, Skeletal muscle atrophy, Calpains, Calpastatin, Intracellular calcium

## Abstract

**Background:**

Skeletal muscle atrophy is the net loss of muscle mass that results from an imbalance in protein synthesis and protein degradation. It occurs in response to several stimuli including disease, injury, starvation, and normal aging. Currently, there is no truly effective pharmacological therapy for atrophy; therefore, exploration of the mechanisms contributing to atrophy is essential because it will eventually lead to discovery of an effective therapeutic target. The *ether*-*a*-*go*-*go related gene* (*ERG1A*) K^+^ channel has been shown to contribute to atrophy by upregulating ubiquitin proteasome proteolysis in cachectic and unweighted mice and has also been implicated in calcium modulation in cancer cells.

**Methods:**

We transduced C_2_C_12_ myotubes with either a human *ERG1A* encoded adenovirus or an appropriate control virus. We used fura-2 calcium indicator to measure intracellular calcium concentration and Calpain-Glo assay kits (ProMega) to measure calpain activity. Quantitative PCR was used to monitor gene expression and immunoblot evaluated protein abundances in cell lysates. Data were analyzed using either a Student’s *t* test or two-way ANOVAs and SAS software as indicated.

**Results:**

Expression of human *ERG1A* in C_2_C_12_ myotubes increased basal intracellular calcium concentration 51.7% (*p* < 0.0001; *n* = 177). Further, it increased the combined activity of the calcium-activated cysteine proteases, calpain 1 and 2, by 31.9% (*p* < 0.08; *n* = 24); these are known to contribute to degradation of myofilaments. The increased calcium levels are likely a contributor to the increased calpain activity; however, the change in calpain activity may also be attributable to increased calpain protein abundance and/or a decrease in levels of the native calpain inhibitor, calpastatin. To explore the enhanced calpain activity further, we evaluated expression of calpain and calpastatin genes and observed no significant differences. There was no change in calpain 1 protein abundance; however, calpain 2 protein abundance decreased 40.7% (*p* < 0.05; *n* = 6). These changes do not contribute to an increase in calpain activity; however, we detected a 31.7% decrease (*p* < 0.05; *n* = 6) in calpastatin which could contribute to enhanced calpain activity.

**Conclusions:**

Human *ERG1A* expression increases both intracellular calcium concentration and combined calpain 1 and 2 activity. The increased calpain activity is likely a result of the increased calcium levels and decreased calpastatin abundance.

## Background

Skeletal muscle comprises approximately 40% of total human body weight and contains 50–75% of all bodily proteins. Skeletal muscle is needed for the production of mechanical energy, body posture, modulation of body temperature, and for generating force and movement. Thus, a certain amount of skeletal muscle tissue is necessary for well-being and a reduction in this tissue could compromise health [1]. Skeletal muscle mass is maintained by a continuous, fluctuating balance between protein degradation and protein synthesis; however, when the rate of degradation increases or the rate of protein synthesis decreases, muscle mass can be lost in a process known as atrophy. Skeletal muscle atrophy is defined as a 5% or greater decrease in muscle mass and strength and can be induced by certain stimuli: muscle disuse, denervation, starvation, disease (e.g., diabetes and cancer), loss of neural input, and even normal aging [2, 3]. Treatments for skeletal muscle atrophy currently under study include administration of pharmaceuticals such as growth factors [4], beta-agonists [5], inhibitors of proteolysis [6, 7], stimulators of protein synthesis [8], and myostatin inhibitors [9–11]; however, these are not adequately effective. Thus, further investigation into the mechanisms resulting in atrophy is needed to reveal new and improved targets for therapy.

The protein degradation that contributes to atrophy occurs mainly through four proteolytic pathways: the ubiquitin proteasome pathway (UPP), cathepsins (the autophagy-lysosome system), caspases (the apoptosis protease system), and calpain enzymes. Calpains are a family of calcium activated cysteine proteases that cleave specific proteins to release large fragments [7]. In skeletal muscle, calpain activity disassembles the sarcomere, releasing actin and myosin to become accessible for ubiquination and subsequent degradation by the proteasome (i.e., the UPP) [12–14]. Indeed, calpains have been shown in vitro to act upon anchoring proteins (e.g., titin, nebulin, and desmin) which attach the sarcomere’s myofilaments to the sarcomeric Z-disc [13]. The cleavage of these proteins subsequently releases α-actinin and thus results in the release of the actin thin filament from the myofibril [13, 14]. Calpains have also been shown to degrade tropomyosin and troponin proteins [13] and, combined with the cleavage of titin, this degradation allows for the removal of the thick filaments from the myofibrils. Calpain activity has also been shown to affect the Akt pathway which modulates the balance of protein synthesis and degradation [14].

The ERG1a (*ether*-*a*-*go*-*go related gene*) gene encodes a potassium channel known to conduct cardiac I_*Kr*_ current and be partially responsible for the repolarization of the heart action potential [15–17]. ERG1 is detected in numerous mammalian tissues including brain and heart, but had not been reported in skeletal muscle until we demonstrated that ERG1a protein abundance increases in the skeletal muscle of mice in response to hind limb suspension and tumor expression [18]. We further showed that, when ectopically expressed in the skeletal muscle of weight bearing mice, ERG1a increases the abundance of the UPP E3 ligase, MuRF1, and overall UPP activity [18]. These data suggest that ERG1a participates in the process of skeletal muscle atrophy at least partially through modulation of the UPP [15]. We hypothesized that ERG1a could affect other proteolytic pathways. Indeed, human ERG1A (HERG1A) has been shown to increase the basal intracellular calcium concentration ([Ca^2+^]i) of SKBr3 breast cancer cells [19] and is detected in the t-tubules of cardiac tissue [17, 20] where it has the potential to affect the calcium release mechanism. Thus, we hypothesized that HERG1A would increase intracellular concentration in C_2_C_12_ myotubes and consequently enhance calpain activity. Here, we describe studies designed to explore this hypothesis and demonstrate that indeed, ERG1A enhances both intracellular calcium concentration and calpain activity.

## Methods and materials

### Antibodies

The following antibodies were used: Calpain-1 polyclonal antibody 3189-30 T (BioVision, Milpitas, CA); Calpain-2 polyclonal antibody 3372-30 T (BioVision, Milpitas, CA); Calpain-3 polyclonal antibody A11995 (ABclonal, Woburn, MA); Calpastatin polyclonal antibody A7634 (ABclonal, Woburn, MA); MF-20 myosin antibody (Developmental Studies Hybridoma Bank, Iowa City, IA); laminin antibody NBP2–44751 from rat (Novus, Centennial, CO); erg1 antibody P9497 (Sigma, St. Louis, MO); and GAPDH polyclonal antibody ABS16 (Sigma, St. Louis, MO).

### Cell culture

C_2_C_12_ myoblasts were grown in Dulbecco’s modification of Eagle’s medium (DMEM) supplemented with 10% fetal bovine serum (FBS) and maintained in a humidified incubator with 10% CO_2_ at 37 °C. To differentiate myoblasts into myotubes, cells were grown in DMEM supplemented with 10% FBS to ~ 85% confluence. The FBS medium was then replaced with DMEM medium supplemented with 2% heat-inactivated horse serum. Cells were incubated for 4 days to allow for terminal differentiation.

### Viral transduction

Terminally differentiated C_2_C_12_ myotubes were treated with 200 MOI virus to produce HERG1A protein after 48 h. Specifically, for experimentation one set of cells was treated with control GFP encoded adeno-virus (VQAd EMPTY-eGFP; ViraQuest, New Liberty, IA) while the other received the same GFP encoded adeno-viral particles also encoding the human ERG1A K^+^ channel (VQAd CMV Herg-GFP; ViraQuest). The cells were then incubated for 48 h and monitored via fluorescence to verify that the transduction was successful.

### Animals

All procedures were approved by the Southern Illinois University Carbondale (SIUC) Animal Care and Use Committee. A total of 80 ND4-Swiss Webster 7–8-week-old male mice (Harlan-Sprague; Indianapolis, IN) were used. Animals were housed in SIUC vivarium facilities on a 12 h light/dark cycle, monitored by lab animal veterinarians, and provided food and water ad libitum*.*

### Western blot

**Fig. 1:**
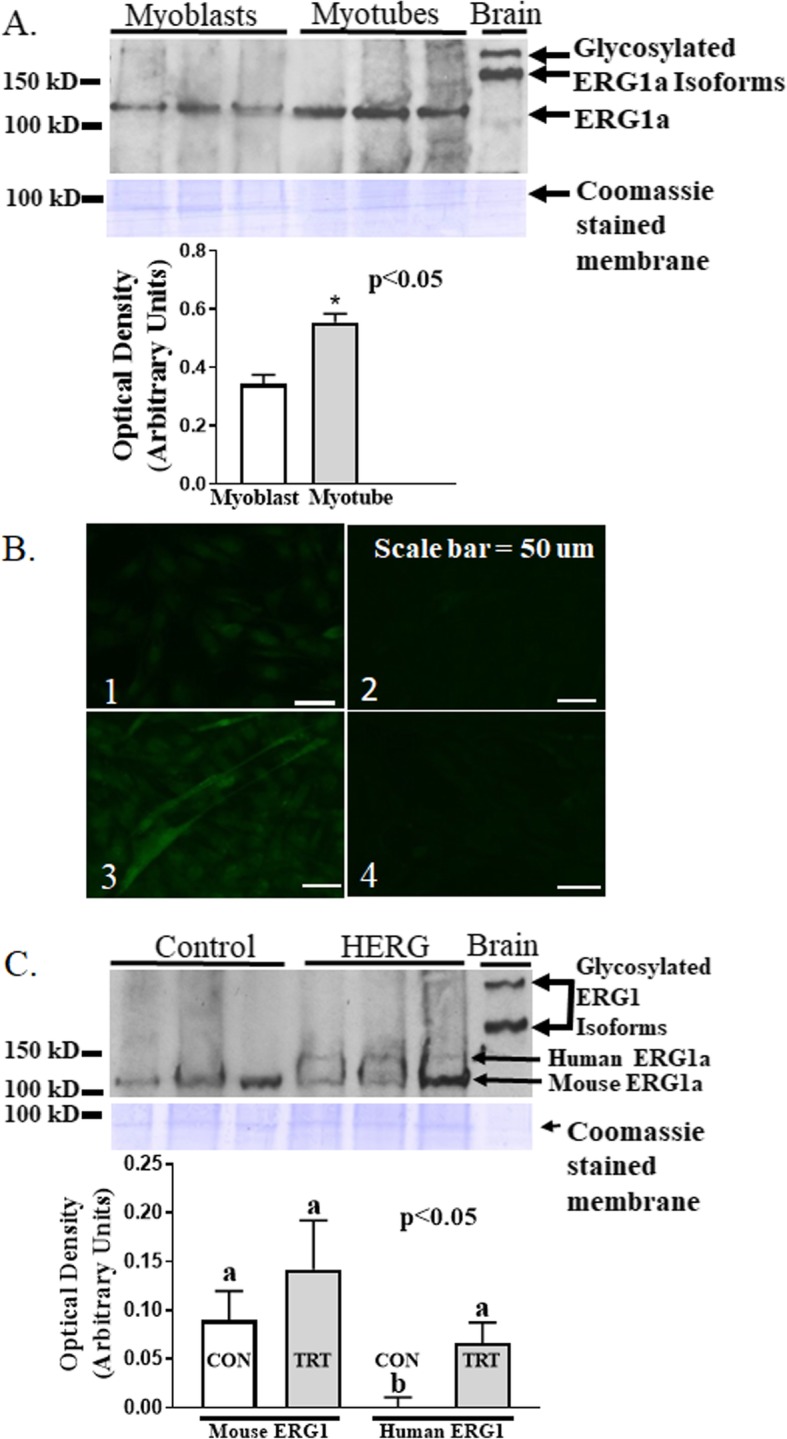
Transduction of C_2_C_12_ myotubes with a HERG1A-encoded adenovirus results in elevated HERG1A protein. **a** Immunoblot of equal protein content (50 μg) from lysates of non-transduced cells reveals that native ERG1 protein is 40.7% (*p* < 0.01; *n* = 6; Student’s *t* test) more abundant in myotubes than in myoblasts. Coomassie stained membrane confirms that equal amounts of cell lysate protein were loaded into each lane. **b** Immunohistochemistry labeling ERG1 protein with Alexfluor 488 (green) secondary antibody confirms that native ERG1 protein is more abundant in myotubes than in myoblasts. Representative images of immune-stained cells: (1) myoblasts immunostained with ERG1 primary antibody; (2) myoblasts immunostained without ERG1 primary antibody as control; (3) myotubes immunostained with ERG1 primary antibody; (4) myotubes immunostained without ERG1 primary antibody as control. Scale bar = 50 μm. **c** Transduction of C_2_C_12_ myotubes with a HERG1A-encoded adenovirus results in synthesis of HERG1A protein as demonstrated by immunoblot (*p* < 0.05; *n* = 6; two-way ANOVA). Coomassie stained membrane (blue) reveals that equal amounts of cell lysate protein were loaded into each lane

**Fig. 2:**
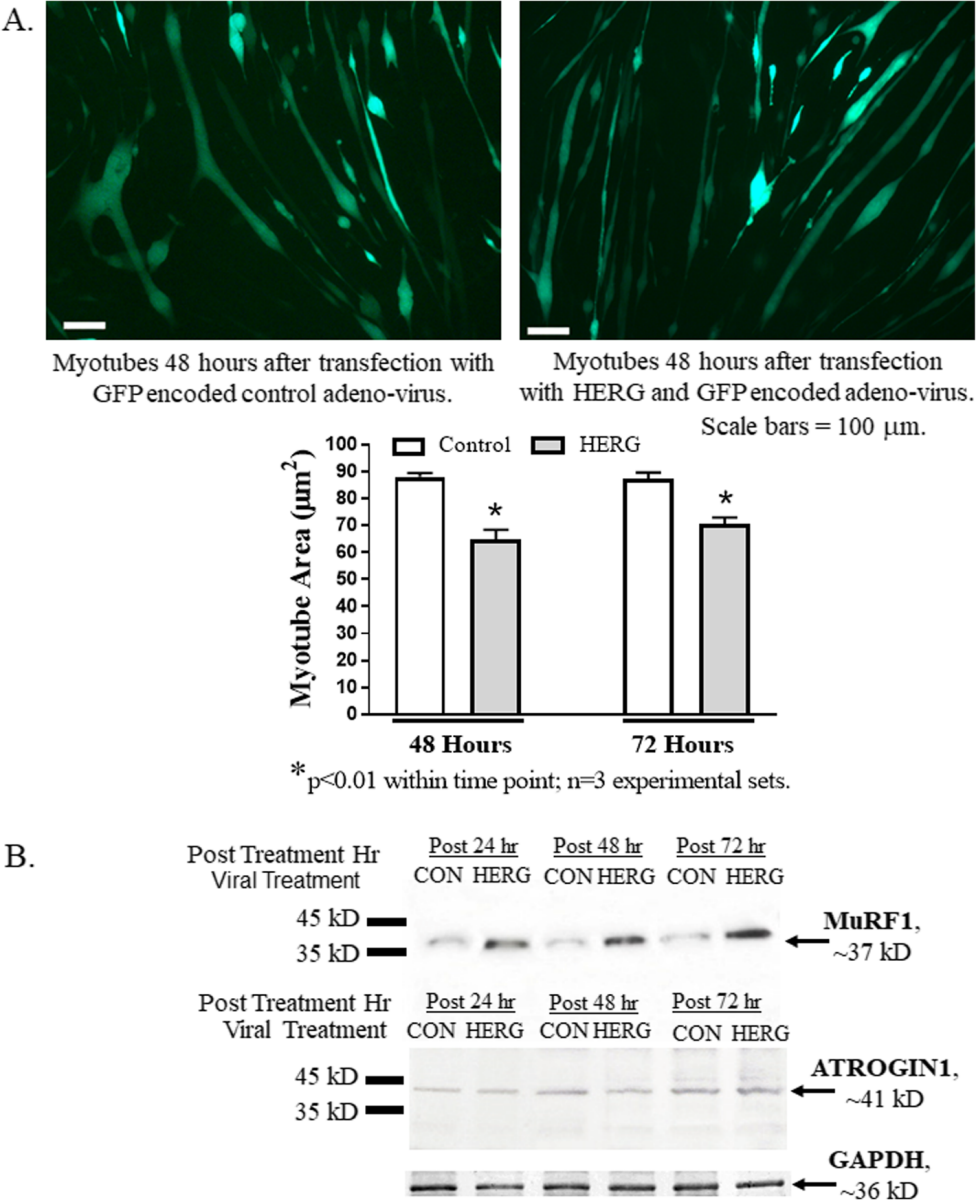
Transduction of myotubes with HERG1A-encoded adenovirus is a valid in vitro skeletal muscle atrophy model. **a** The area of myotubes treated with HERG1A-encoded adenovirus is a significant 26.4% smaller (*p* < 0.01; *n* = 3 experimental sets) than that of control myotubes at 48 h after transduction and a significant 19.3% smaller (*p* < 0.01; *n* = 3 experimental sets) at 72 h after transduction. Scale bar = 100 μm. Bars of the graph represent the mean myotube area (μm^2^) while the error bars represent the standard error of the mean. **b** Immunoblot shows that transduction of C_2_C_12_ myotubes with a HERG1A-encoded adenovirus yields an early increase in MuRF1 E3 ligase protein abundance while it does not increase abundance of ATROGIN1 protein. Immunoblots are representative of three experiments

Membrane proteins were extracted from C_2_C_12_ myoblasts and myotubes for Fig. 1a and from C_2_C_12_ myotubes at 48 h after viral transduction for Figs. 1, 5, 6, 7, and 8c, b, b. Membrane proteins were extracted from C_2_C_12_ cells using Tris buffer (10 mM, pH 7.4) containing 1 mM EDTA, 2% Triton X-100, and protease inhibitors (0.5 mM pefabloc, 0.5 mM PMSF, 1 mM benzamidine, 1 mM pepstatin, and 1 mM 1,10-phenanthroline). Samples were triturated using a tuberculin syringe and 23G needle and allowed to incubate on ice at 4 °C for 30 min and then centrifuged for 2 min at 15,000 rpm. Cellular proteins for Fig. 2b were extracted from C2C12 myotubes at 24, 48, and 72 h after transduction using Tris buffer (10 mM, pH 7.4) containing 1 mM EDTA, and protease inhibitors (0.5 mM pefabloc, 0.5 mM PMSF, 1 mM benzamidine, 1 mM pepstatin, and 1 mM 1,10-phenanthroline). The samples were then centrifuged for 2 min at 15,000 rpm. All supernatants were collected and the protein content was determined using a DC protein assay kit (BioRad, Hercules, CA) and manufacturer’s instructions. Samples were electrophoresed through a 4% poly-acrylamide stacking gel followed by a 7.5% poly-acrylamide separating gel and finally transferred to PVDF membrane (BioRad, Hercules, CA). Membranes were immunoblotted using one or more of the antibodies listed above and developed with Immun-Star AP chemiluminescent substrate (BioRad, Hercules, CA). Optical densities of the protein bands were determined using ImageJ software (NIH).

### Fusion index

Myoblasts were grown on glass coverslips coated with rat tail collagen and then treated with either the HERG-encoded or the control virus and allowed to terminally differentiate. These were then immunostained for myosin using the DSHB antibody recognizing myosin and a mouse on mouse (M.O.M.) Kit (Vector Labs, Inc.; Burlingame, CA) per manufacturer’s instructions. The coverslips were then mounted to slides with a mounting substance containing DAPI, and images were acquired using a Leica DM4500 microscope with a Leica DFC 340FX camera. The nuclei of myosin-positive cells were counted in three fields from ten slides (five treated with HERG-encoded virus and five treated with control virus).

### Resting intracellular Ca^2+^ assay

C2C12 myoblasts were cultured in DMEM supplemented with 10% FBS and 1% P/S and plated at a density of 5 × 10^4^ cells/well in a black-walled 96-well plates (Corning Life Sciences). Once myoblasts reached 80–90% confluency, culturing media was exchanged for differentiation media (DMEM supplemented with 2% horse serum and 1% P/S) to promote differentiation and fusion of myoblasts into myotubes. Myoblasts were differentiated for 3–4 days (2–3 days prior to a decrease in myotube viability within a 96-well plate), and the differentiation media was exchanged daily. Using a multiplicity of infection of 100 (based on the initial number of myoblasts plated), myotubes were transduced with adenovirus encoding EGFP control or HERG. Myotubes were grown for two additional days, and the differentiation media was refreshed daily. Prior to Ca^2+^ measurements, the media was removed and myotubes were washed twice with 200 μL PBS. Then, 5 μM Fura2-AM (Molecular Probes, Eugene, OR) was diluted in Krebs-Ringer HEPES buffer (KRBH), and each well of myotubes was incubated in 100 μL of this solution for 1 h at RT. KRBH contained 134 mM NaCl, 3.5 mM KCl, 1.2 mM KH_2_PO_4_, 0.5 mM MgSO_4_, 1.5 mM CaCl_2_, 5 mM NaHCO_3_, and 10 mM HEPES and was supplemented with 0.05% fatty-acid free BSA (pH 7.4). After this period, the Fura2-AM was removed, and myotubes were washed twice with KRBH. Lastly, myotubes were equilibrated in KRBH for 30 min at RT. Fura2 fluorescence was monitored every 0.7 s for a total of 15 s using a Synergy 4 Multimode Microplate Reader (BioTek Instruments, Winooski, VT). Fura2 was excited using a 340/20 nm band-pass excitation filter or 380/20 nm band-pass excitation filter, and emission was collected in both cases using a 508/20 nm band-pass emission filter. The 340/380 nm ratio at each time point was calculated by dividing the Fura2 signal collected at 340 nm by 380 m, and these data points were averaged to yield a resting 340/380 nm ratio, or resting Ca^2+^ level, for each well of myotubes. Seven independent calcium measurements were performed, with each experiment containing between six and 16 replicates, and the average 340/380 nm ratio ± SE was calculated among all wells for GFP- and HERG-transduced myotubes.

### Quantitative real time PCR

Total RNA was extracted from C_2_C_12_ myotubes using Trizol reagent (Life Technologies; Carlsbad, CA) according to manufacturer’s instructions followed by chloroform solubilization and ethanol precipitation. Contaminating DNA was degraded via DNase (RQ1 RNase-Free DNase; ProMega, Madison WI). The total RNA was then reverse transcribed using a GOScript™ Reverse Transcription System Kit (Promega) per manufacturer’s instructions. Quantitative PCR was then performed using PowerUp SYBR green master mix (Applied Biosystems, Foster City, CA) and primers for the gene of interest along with primers for the 18S ribosomal subunit “housekeeping gene” (Table 1). An Applied Biosystems 7300 real-time PCR system was used to detect SYBR green fluorescence as a measure of amplicon. Changes in gene expression were determined using the Livak method to normalize the gene of interest to the “housekeeping gene.”

**Table 1:** Sequences of primers used for quantitative PCR

Primer name (mouse)	Primer sequence 5′–3′	Size (bp)^a^	Tm (°C)	GC (%)	Amplicon size (bp)^a^
Merg1a forward	cctcgacaccatcatccgca	20	59.6	55.0	145
Merg1a reverse	aggaaatcgcaggtgcaggg	20	60.3	60.0	
18S subunit forward	cgccgctagaggtgaaattct	21	57.2	52.4	101
18S subunit reverse	agaacgaaagtcggaggttc	20	57.0	52.4	
Calpain 1 forward	gctaccgtttgtctagcgtc	20	58.73	55.0	98
Calpain 1 reverse	taactcctctgtcatcctctggt	23	59.99	47.83	
Calpain 2 forward	ttttgtgcggtgtttggtcc	20	59.83	50.0	107
Calpain 2 reverse	aactcagccacgaagcaagg	20	60.89	55.0	
Calpain 3 forward	ttcacaggaggggtgacaga	20	60.11	55.0	122
Calpain 3 reverse	ttcgtgccatcgtcaatggag	21	61.01	52.38	
Calpastatin forward	gccttggatgacctgataga	20	53.8	50.0	115
Calpastatin reverse	gtgcctcaaggtaggtagaa	20	53.7	50.0	

^a^*bp* base pair

### Tissue sections and immunohistochemistry

For Fig. 4, mouse *Gastrocnemius* muscles were embedded in OCT, cryo-sectioned (20 μm), and stained for β-galactosidase (lacZ) activity as described earlier [18]. Sections for immunohistochemistry were fixed in cold methanol at − 20 °C for 10 min. These were then rinsed with PBS at room temperature (RT) and incubated in 3% H_2_O_2_ for 1 h. These were then rinsed thoroughly in PBS and incubated with blocking reagent I (10% normal goat serum [NGS], 0.1% bovine serum albumin [BSA; Sigma, St. Louis, MO], and 0.1% Tween-20 in PBS) for 1 h at RT. The slides were then incubated for one hour with the laminin antibody (2 μg/mL in blocking reagent II–5% NGS and 0.2% TritonX100 in PBS) or in blocking reagent II only as a control for primary antibody binding. After a thorough rinsing with PBS, the slides were incubated overnight in the erg1 antibody (1:10 in blocking reagent 2) or in blocking reagent 2 alone on the control sections. The next day, the sections were rinsed thoroughly in PBS containing 0.1% Tween-20. All sections were then incubated for 1 h at RT in Alexafluor 568 goat anti-rat IgG (1:1000 in blocking reagent II) to bind the laminin primary antibody from rat. The slides were then again rinsed with PBS and incubated for one hour at RT in the goat anti-rabbit secondary antibody from the Alexafluor 488 Tyramide Super Boost Kit (Invitrogen, Carlsbad, CA). The tyramide reaction was carried out per manufacturer’s instructions to identify ERG1 protein with green fluorescence. Finally, the sections were rinsed thoroughly with PBS and mounted with Fluoromount G with DAPI (EMS; Hatfield, PA). Two sections from each muscle mid-section were analyzed.

### Imaging

Images were acquired using a Leica DM4500 microscope with a Leica DFC 340FX camera. Acquisition parameters were maintained identically across samples to allow for comparison of immunofluorescence levels when these comparisons were made. For assay of laminin protein fluorescence, two fields were imaged per slide (one slide per mouse) and the single point brightness was measured for 50 random consecutive points within the sarcolemma of each complete fiber within each field using ImageJ [21] and methods adapted from those published previously [22]. Brightness values were recorded as integers ranging from 0 (no signal) to 256 (white). The average brightness value (± standard error of the mean, SEM) for each section was determined and analyzed by two-way ANOVA using the General Linear Model Procedure of SAS 9.4 (SAS Institute Inc., Cary, NC).

### Plasmids

The mouse *Erg1a* (*Merg1a*) clone in pBK/CMV plasmid [23] was a generous gift from Dr. Barry London (Cardiovascular Institute, University of Pittsburgh, PA). The phRL synthetic *Renilla* luciferase reporter vector was purchased from ProMega (Madison, WI).

### Electro-transfer

Mouse anesthesia was induced with 4% isoflurane in a vented chamber and maintained by administration of 2.5% isoflurane in oxygen using a properly ventilated nose cone with anesthesia machine and scrubber. Once the animals were well anesthetized, the hind limbs were shaved and the *Gastrocnemius* muscles were injected with expression plasmids in 50 μL sterile saline and then stimulated with 8 pulses at 200 V/cm for 20 ms at 1 Hz with an ECM 830 ElectroSquare Porator (BTX; Hawthorne, NY). This method has been shown to result in ERG1a protein synthesis in skeletal muscle [15, 18].

### Animal study design

#### Study 1

The *Merg1a* plasmid (30 μg) and a plasmid encoding *Renilla* reporter (5 μg) were injected into the left *Gastrocnemius* muscles of mice (*n* = 40). An empty control plasmid (30 μg) and the *Renilla* reporter plasmid (5 μg) were injected into the *Gastrocnemius* muscles of the right legs. All legs were electro-transferred to improve plasmid uptake and expression. Each day, at days 0–7, five mice were humanely killed and the *Gastrocnemius* muscles were harvested and frozen immediately in liquid nitrogen. These were then stored at − 80 °C. All muscles were later thawed, homogenized, and assayed for (1) protein content, (2) *Renilla* activity to determine transfection efficiency, and (3) calpain activity.

#### Study 2

The *Gastrocnemius* muscles of a second set of animals, consisting of five animals per day for days 0–5 and 7 (*n* = 35), were injected and electro-transferred as described above. After the appropriate amount of time, the animals were humanely sacrificed, the muscles were harvested, and total RNA was extracted for rtPCR assay.

#### Study 3

The *Merg1a* plasmid (30 μg) and a plasmid encoding a β-galactosidase (LacZ) reporter (5 μg) were injected into the left *Gastrocnemius* muscles of mice (*n* = 5). An appropriate empty control plasmid (30 μg) and the LacZ reporter plasmid (5 μg) were injected into the *Gastrocnemius* muscles of the right legs. All legs were electro-transferred to improve plasmid uptake and expression. At day 5, the five mice were humanely killed and the *Gastrocnemius* muscles were harvested and frozen immediately in liquid nitrogen. These were then stored at − 80 °C. All muscles were later thawed and painstakingly serially sectioned. Serial sections were then stained for either lacZ or dually immunostained for MERG1 and laminin proteins as described above.

### Protein assay

The BCA D/C Protein Assay Reagents (BioRad; Carls Bad, CA) were used to assay both samples and standards (0, 0.25, 0.5, 1.0, 1.25, 1.5, 2.0 mg/mL bovine serum albumin in Passive Lysis Buffer [ProMega; Madison, WI]) for protein content, using a Synergy H1 Hybrid Reader (BioTek; Winooski, VT) to measure absorbance at 605 nm light wavelength. Sample absorbances were interpolated against the standard curve to determine the protein concentration of each sample.

### *Renilla* activity

To control for differences in transfection efficiency in the animal muscle, a plasmid encoding the *Renilla* luciferase enzyme was electro-transferred into muscle along with the *Merg1a* plasmid (as described above). The *Renilla*-Glo™ Luciferase Assay System (ProMega) was used, according to manufacturer’s instructions, to assay homogenates for *Renilla* enzyme activity. The reaction was allowed to proceed for the recommended 10 min and luminescence was measured using a Synergy H1 Hybrid Reader (BioTek; Winooski, VT). Luminescence was measured again 10 min later to ensure that the reaction had reached an end point after the first 10 min. The data are reported in relative light units (RLU).

### Calpain assay

A Calpain-Glo Kit (ProMega; Madison, WI) was used to determine calpain activity in both myotubes and mouse muscle.

#### Myotubes

Myotubes were terminally differentiated and then transduced with either a HERG1A-encoded adeno-virus or the same (but non-HERG1A-encoded) virus as control (12 wells each). At 48 h post-transduction, wells were washed with two changes of 37 °C PBS and then PBS (200 μL) containing 0.2% Triton X-100 and 200 nM epoxomicin (BostonBiochem, Cambridge, MA, Cat. #I-110) was added to permeabilize the cells and to inhibit the proteasome, respectively. Six wells per viral treatment (HERG1A or control) received the buffer described (i.e., native activity); however, six wells per viral treatment received buffer supplemented with the calpain inhibitor MDL28170 (50 μM). These were allowed to sit at room temperature for 5 min to ensure the myotubes were permeabilized and the inhibitors had taken effect. Then 200 μL of Calpain-Glo reagent was added to all wells, mixed gently, and allowed to sit at room temperature. After 15 min, a 200 μL aliquot of the liquid was removed from each well and placed in a white-walled 96-well plate and luminescence was read using a Synergy H1 Hybrid Reader (BioTek Instruments, Winooski, VT). The remaining well contents were scraped from the back of the plate, triturated using a syringe and 26 gauge needle, and then centrifuged (13,000×*g*; 3 min) to remove any solid material. The supernatant was assayed for protein content using the BioRad DC Protein Assay kit. The protein data were used to normalize the calpain RLU activity.

#### Mouse muscle samples

The *Gastrocnemius* muscles were thawed, weighed, and homogenized in Passive Lysis Buffer (PLB; ProMega) at a concentration of 2.5 μL buffer/μg tissue. The sample homogenates were aliquoted and frozen at − 80 °C. Prior to assay, the homogenates were thawed and sample aliquots (40 μL) and positive control (purified porcine calpain) were added to wells of 96-well plates with assay buffer (40 μL) having either 2 mM calcium (to activate calcium dependent enzymes) or 2 mM calcium plus 50 mM MDL28170 (to inhibit calpain specifically while allowing other calcium activated enzymes to function). Each 96-well plate was read with a Synergy H1 Hybrid Reader (BioTek; Winooski, VT) and activity was measured in RLU. Calpain activity was determined by subtracting the RLU of the wells treated with 2 mM calcium and MDL28170 from the RLU of the wells treated with 2 mM calcium only and normalizing this RLU to the RLU assayed with the *Renilla* kit to control for differences in transfection efficiencies. The result was then normalized to protein content (RLU/mg protein).

### Statistics

In general, statistics were done using either a simple Student *t* test or an ANOVA (as indicated in results section and figure legends) and SAS (SAS Inc.; Carey, NC). Results were considered significant when *p* < 0.05 unless otherwise noted.

## Results

### Transduction of C_2_C_12_ myotubes with a HERG1A-encoded adenovirus results in elevated HERG1A protein

Immunoblot of equal protein aliquots from both non-virus treated C_2_C_12_ myoblast and myotube lysates detects a 40.7% (*p* < 0.01; *n* = 6; Student’s *t* test) greater abundance of the ERG1 protein in myotubes than in myoblasts (Fig. 1a). Immunohistochemistry work also demonstrates that there is more ERG1 protein in the C_2_C_12_ myotubes than in the myoblasts, revealing a stronger signal in myotubes that is dispersed over the surface of the cell, while in myoblasts it reveals only a very faint fluorescent signal which appears to be mainly nuclear (Fig. 1b). We transfected myotubes with either virus-encoding HERG1A (and GFP) or with the same, but not HERG1A-encoded, virus as control. Immunoblot of the lysates shows that C_2_C_12_ myotubes transfected with virus encoding HERG1A do synthesize the HERG1A protein, which appears as a single band of higher mass (likely a result of differential glycosylation) than the native mouse ERG1 and is absent from the myotubes treated with the control virus (Fig. 1c; *p* < 0.05; two-way ANVOA). Coomassie stained membrane confirms that equal amounts of protein were loaded into each well of the gel for immunoblot.

### Transduction of C_2_C_12_ myotubes with a HERG1a-encoded adenovirus results in decreased myotube area and increased MuRF1 E3 ligase abundance, but no change in myoblast fusion index

We transfected myotubes with either virus-encoding HERG1A (and GFP) or with the same, but not HERG1A-encoded, virus as control. Fluorescent imaging demonstrates that both viral particles infect myotubes (Fig. 2a). Further, when the average area (μm^2^) of fluorescent myotubes from both sets is determined at both 48 and 72 h after transfection, we discover that, similarly to mouse skeletal muscle fibers electro-transferred with *Merg1a* plasmid [23], the myotubes transfected with HERG1A are significantly smaller than control myotubes. Specifically, the area of the HERG1A-expressing myotubes is decreased by 26.4% at 48 h post transfection (*p* < 0.01; *n* = 6; Student’s *t* test) and by 19.3% at 72 h post transfection (*p* < 0.01; *n* = 6; Student’s *t* test). Within each time point, the difference between the HERG1A-treated and control myotubes is statistically significant (*p* < 0.01); however, there is no significant difference in size between the myotubes treated with HERG1A-encoding virus at the two different time points (Fig. 2a). Also similarly to mouse skeletal muscle expressing *Merg1a* [23], myotubes transduced with HERG1A exhibit increased levels of the UPP E3 ligase, MuRF1, but not the E3 ligase ATROGIN1 (Fig. 2b). However, when we treated myoblasts with either the HERG-encoded or the control virus and allowed them to differentiate, we found that the HERG-expressing samples did not have a significantly different number of myotubes containing two or more nuclei than the cells treated with the control virus. That is, the fusion index (myosin-positive multi-nucleated cells:total myosin-positive cells evaluated) was 33.5 ± 5.0% (mean ± SEM) for the cells treated with the HERG-encoded virus while it was 31.6 ± 2.3% for the control-treated myoblasts (*p* < 0.74; *n* = 14; Student’s *t* test). The data demonstrate that HERG1A treatment of myotubes results in atrophy (i.e., reduced myotube area) as it does in mouse skeletal muscle; however, it does not affect the degree to which the myoblasts fuse. We conclude that we have developed a valid in vitro model of skeletal muscle atrophy.

### Transduction of myotubes with a HERG1A-encoded adenovirus yields a basal increase in both intracellular calcium levels and calpain activity

**Fig. 3:**
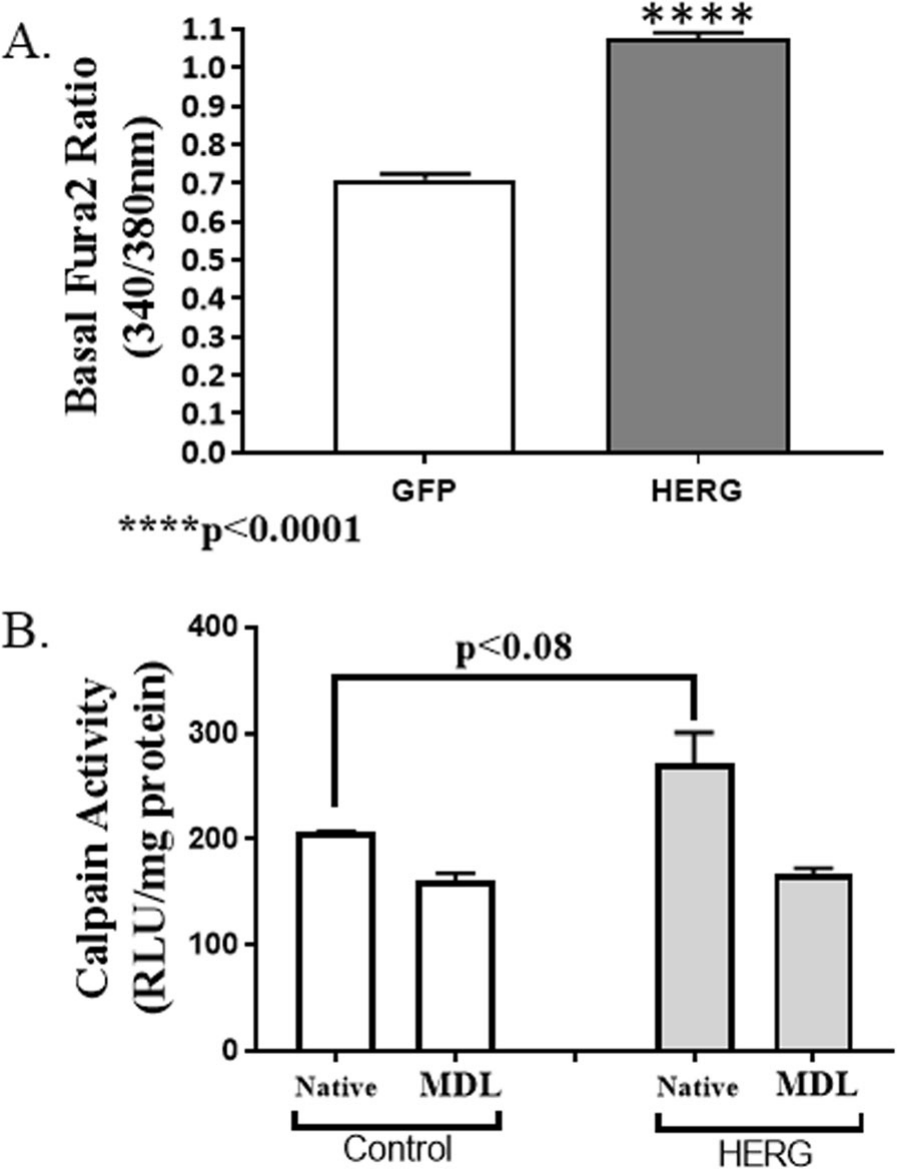
Transduction of myotubes with HERG1A-encoded adenovirus increases basal intracellular calcium levels and basal calpain activity. **a** Fura-2 dye experiments reveal that expression of HERG1A in C_2_C_12_ myotubes yields a 51.9% increase (*p* < 0.0001; *n* = 90 GFP and *n* = 87 HERG1A transduced wells) in basal intracellular calcium levels relative to myotubes transduced with a control virus. **b** Calpain assay reveals that transduction of C_2_C_12_ myotubes with a HERG1A-encoded adenovirus increases combined native calpain 1 and 2 activity a significant 31.9% (*p* < 0.08; *n* = 24; two-way ANOVA) over control myotubes. All bars represent the mean while error bars represent the standard error of the mean

We transduced C_2_C_12_ myotubes with either a GFP- and HERG1A-encoded adenovirus or an appropriate control GFP-only encoded adenovirus. At 48 h after viral treatment, we used a fura-2 calcium indicator assay and observed a significant 51.7% increase (*p* < 0.0001; *n* = 90 GFP and *n* = 87 HERG1A transduced wells; Student’s *t* test) in basal intracellular calcium levels in HERG1A transduced myotubes relative to control (Fig. 3a). This demonstrates that HERG1A must either increase calcium influx and/or intracellular calcium release and/or decrease intracellular calcium re-uptake. Because HERG1A transduction results in increased basal intracellular calcium levels, we investigated the downstream effects of this increase. Specifically, using a Calpain-Glo assay kit (ProMega), we measured the combined activity of the calpain 1 and 2 enzymes in myotubes treated with either the control or the HERG1A-encoded virus. Some myotubes from both viral treatments were treated with either 50 μM MDL28170 to inhibit calpains or an equal volume of buffer vehicle. We observed that basically the same amount of enzyme activity (control myotubes = 160.8 ± 7.3 and HERG1A-expressing myotubes = 167.5 ± 5.34 RLU/mg protein; *n* = 24) was not blocked in each well treated with the MDL28170, indicating that a rather high level of non-calpain activity was assayed. Nonetheless, we find that in control cells, the calpain activity is 22.1% of the total native activity while it is 38.5% of the total in HERG1A-treated cells, demonstrating an increase in calpain activity in the HERG1A-treated cells. Because a two-way ANVOA reveals there is no real difference in the level of MDL28170 inhibited activity, we can compare the differences in assayed native activity (control versus HERG1A treated) and find that there is a 31.9% increase (*p* < 0.08) in activity in the HERG1A-expressing myotubes over the controls (Fig. 3b). Although the 0.08 probability is greater than the generally accepted statistical significance level of 0.05, we believe that the difference is nonetheless real.

### *Merg1a* expression in mouse *Gastrocnemius* muscle increases calpain activity, but did not change the number of centrally located nuclei or laminin abundance

**Fig. 4:**
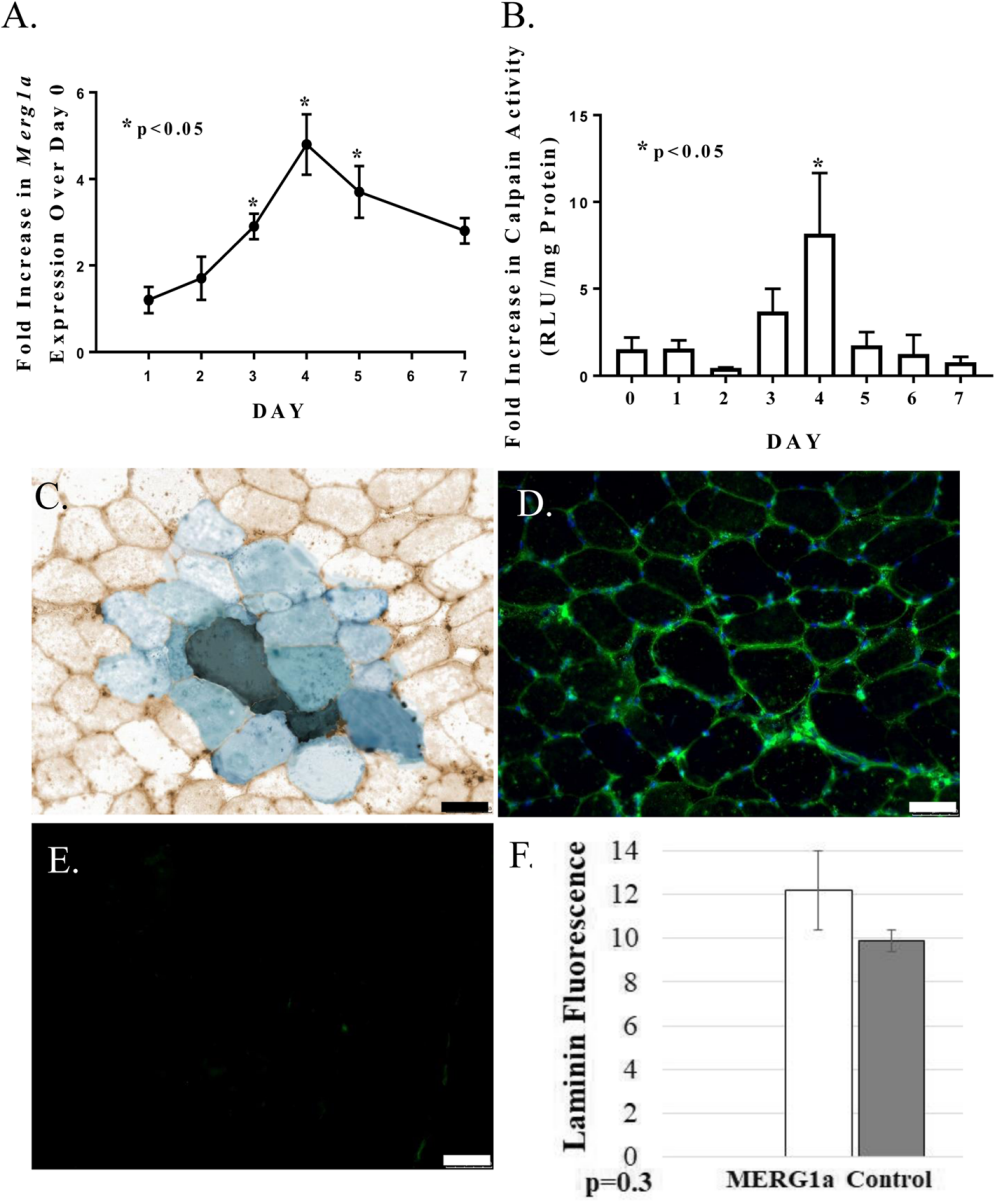
Expression of mouse *erg1a* in mouse *Gastrocnemius* muscle increases *Merg1a* transcription and native calpain activity, but does not increase the number of centrally located nuclei or the abundance of laminin protein. **a** Quantitative PCR shows that electro-transfer of an expression plasmid encoding mouse *erg1a* (*Merg1a*) into mouse skeletal muscle produces *Merg1a* expression which is significantly higher than day 0 at days 3–5 (*p* < 0.05; *n* = 28). The enclosed circles of the line graph represent the mean while the error bars represent the standard error of the mean. **b** *Merg1a* transfection in mouse skeletal muscle increases calpain activity nearly 4-fold (over day 0) by day 3 and nearly 7.5-fold by day 4 (*p* < 0.05; *n* = 40). It returns to day 0 control levels by day 5 post transfection. Bars represent the mean calpain activity while error bars represent the standard error of the mean. **c** Positive assay for the β-galactosidase reporter (as an indicator of electro-transfer of plasmid encoding the Merg1a gene) produces a blue color. **d** Immunostain for MERG1 (green) of a serial section matched to the section in **c** demonstrates that there is indeed a greater amount of MERG1 in the fibers colored blue in **c**. There were no greater number of centrally located nuclei in the green fibers of any sections (*n* = 5 mice). **e** Representative of sections immunostained without primary antibody. **f** Over-expression of *Merg1a* does not produce a change in laminin abundance (*p* = 0.3; *n* = 5). Bars represent the mean single point laminin fluorescence while error bars represent the standard error of the mean. All scale bars = 50 μm

To test the effect of *Merg1a* expression on calpain activity in animals, we electro-transferred the left *Gastrocnemius* muscle of mice with an expression plasmid encoding *Merg1a* and the right leg muscle with an appropriate control plasmid (*n* = 68 mice). We then assayed total RNA extracted from the muscles for *Merg1a* expression (*n* = 28) and the muscle homogenates for calpain activity (*n* = 40). Quantitative PCR reveals that the electro-transfer did produce *Merg1a* expression which was significantly higher than day 0 at days 3–5 (*p* < 0.05; Student’s *t* test was used to compare each day to day 0; Fig. 4a). *Merg1a* expression also yielded an increase in calpain activity, increasing nearly 4-fold (over day 0) by day 3 and 7.5-fold by day 4 (*p* < 0.05; Student’s *t* test was used to compare each day to day 0; Fig. 4b). It returns to day 0 control levels by day 5. Thus, we show that MERG1a overexpression increases calpain activity and thus protein degradation. It is possible that the increase in intracellular calcium could lead to myofiber degeneration. Thus, we electro-transferred left mouse *Gastrocnemius* muscle with a *Merg1a*-encoded plasmid and a Lac-Z-encoded plasmid while expressing lacZ-encoded plasmid and a an appropriate control plasmid in the right *Gastrocnemius* muscle and performed studies to determine if over-production of this protein would bring about changes indicative of degeneration, specifically changes in the number of centrally located nuclei or in the abundance of basal laminin. Thus, we *painstakingly* stained muscle serial sections for lacZ (Fig. 4c) as a marker for MERG1 and dually immunostained matching serial sections for both MERG1 (green fluorescence, Fig. 4d) and laminin (red fluorescence, not shown) and used a DAPI containing immunomount to identify nuclei (Fig. 4d). There was no response in sections not stained with primary antibody (Fig. 4e). The lacZ stain (blue fibers in Fig. 4c) identifies where the MERG1 overexpression occurs. We find no evidence of any changes in the number of centrally located nuclei (Fig. 4d) nor in the amount of laminin fluorescence (Fig. 4f) in the fibers overexpressing MERG1 in any of the five mice examined nor have we seen any evidence of these occurrences in any of our past studies.

### HERG1A expression in myotubes does not affect expression of calpains 1–3 or calpastatin although it does affect certain protein abundances

**Fig. 5:**
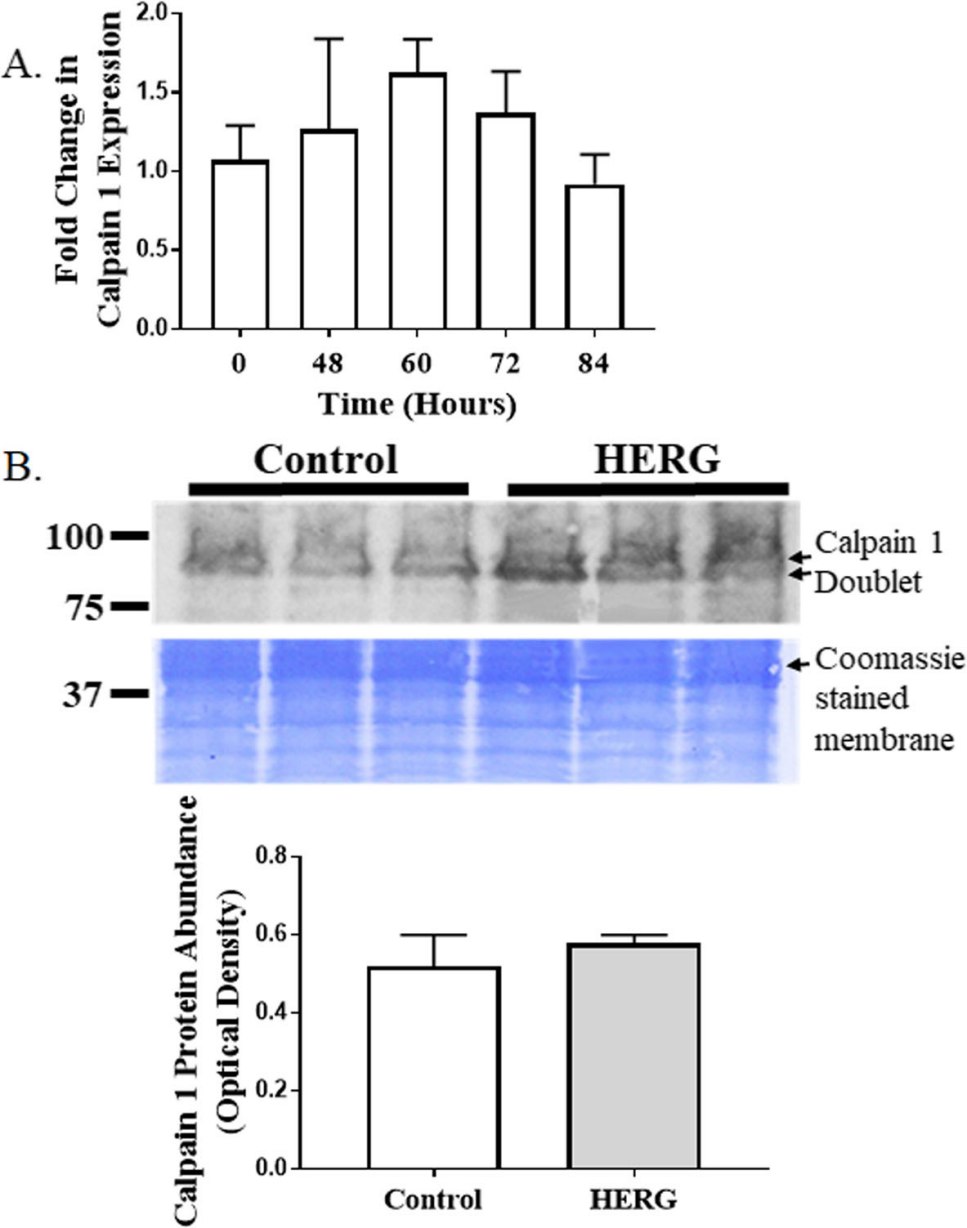
Neither calpain 1 expression nor protein abundance changes after transduction of myotubes with HERG1A-encoded adenovirus. **a** Quantitative PCR reveals that there is no change in expression of calpain 1 for up to 84 h after transduction (*n* = 15). **b**Immunoblot demonstrates that there is no significant change in calpain 1 protein abundance at 48 h after viral transduction (*n* = 6). Bars represent the mean and the error bars represent the standard error of the mean. Coomassie staining of the blotted membrane shows that equal amounts of protein were loaded into each well of the gel

**Fig. 6:**
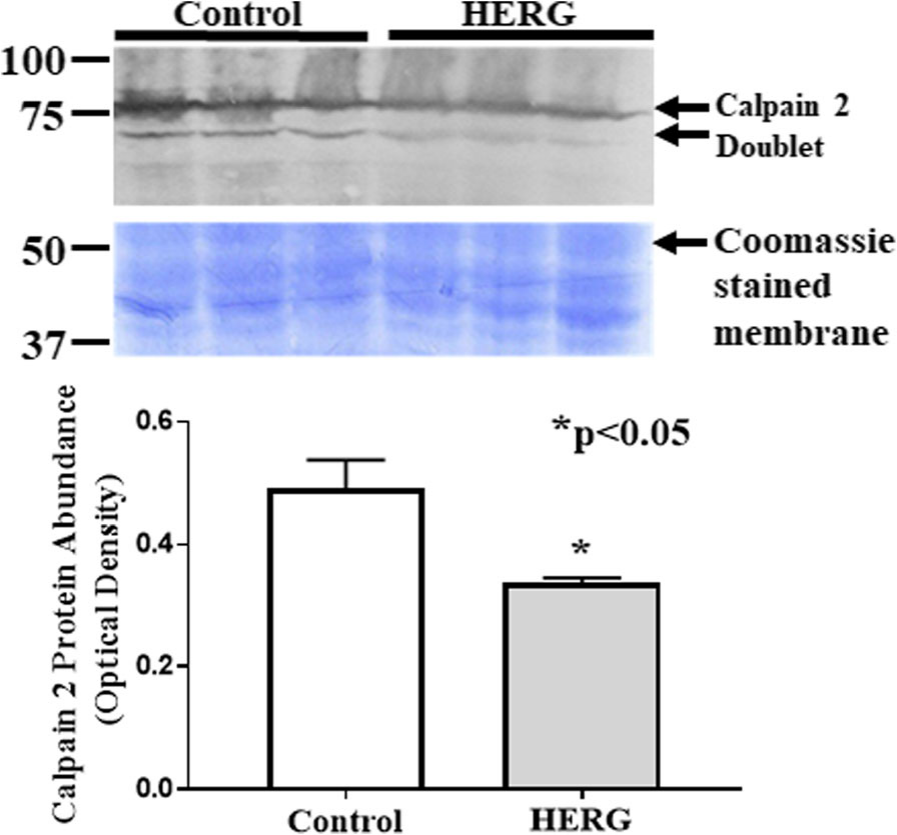
Calpain 2 protein abundance decreases (*p* < 0.05; *n* = 6) 48 h after myotube transduction with HERG1A-encoded adenovirus. Bars represent the mean and error bars represent the standard error of the mean. Coomassie staining of the blotted membrane confirms that equal amounts of protein were loaded into each well of the gel

**Fig. 7:**
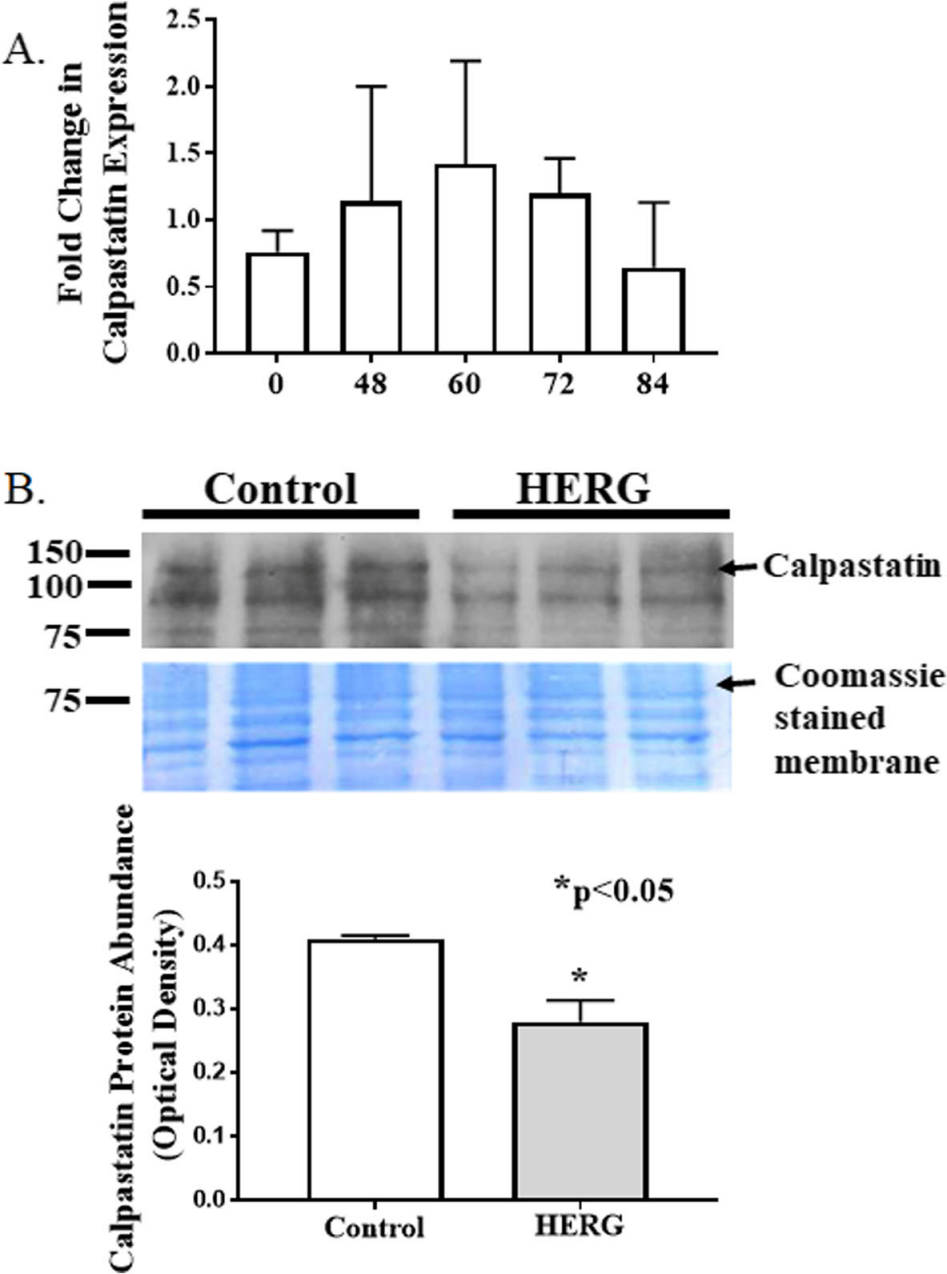
Calpastatin expression does not change after transduction with HERG1A-encoded adenovirus although protein abundance decreases. **a** Quantitative PCR reveals that levels of calpastatin mRNA do not significantly change for up to 84 h after viral transduction with HERG1A encoded adenovirus. **b**, **c**. Immunoblot detects a significant 31.7% decrease in protein abundance (*p* < 0.05; *n* = 6) at 48 h after transduction. All bars represent the mean ± the standard error of the mean. Coomassie staining of the blotted membrane confirms that equal amounts of protein were loaded into each well of the gel

**Fig. 8:**
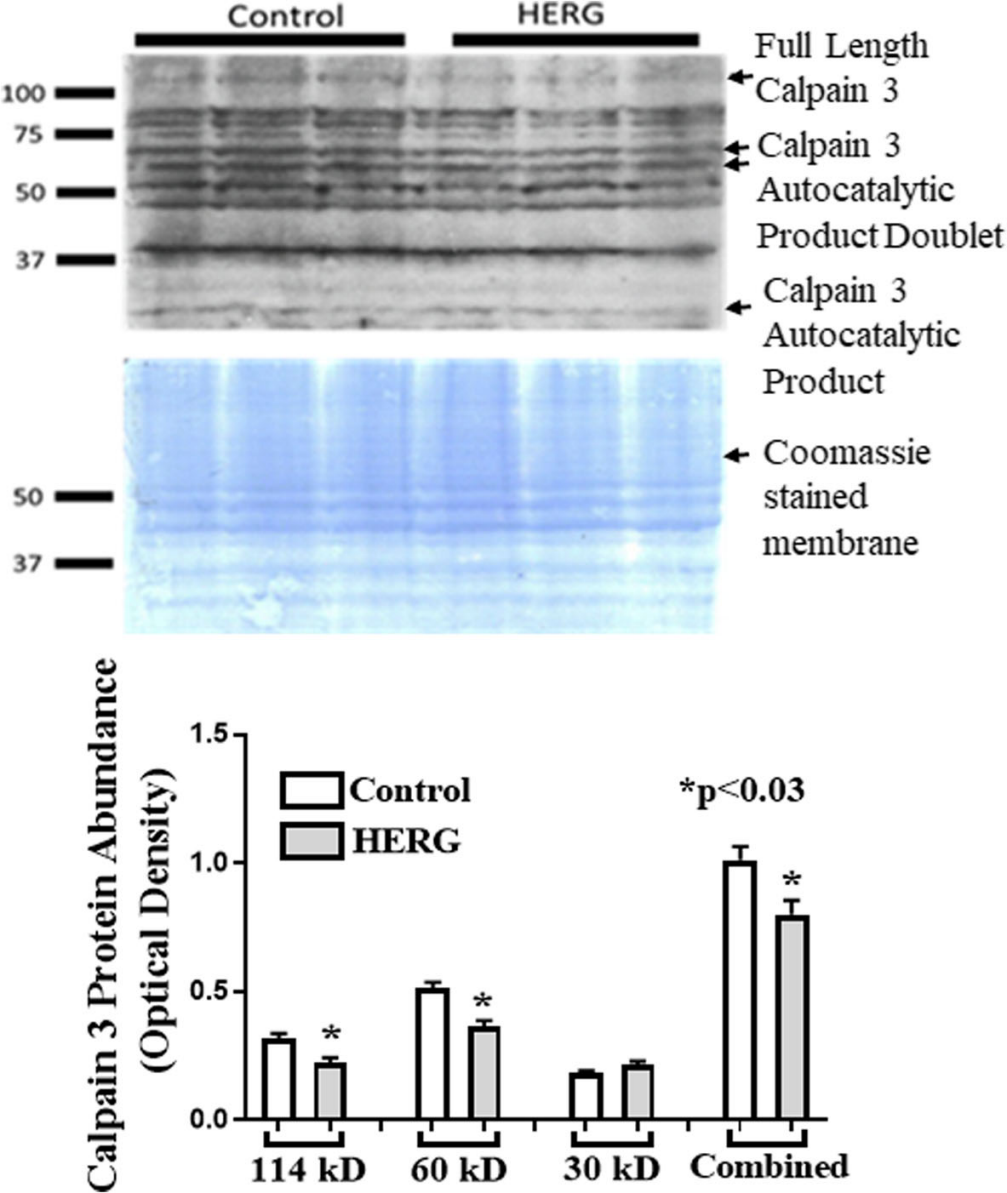
Calpain 3 protein abundance decreased 21.0% in response to transduction of myotubes with HERG1A-encoded adenovirus. Immunoblot shows that calpain 3 degraded into numerous fragments as expected, including three notable autocatalytic products: 114 kD (down 29.6%), 60 kD (down 29.2%), and 30 kD which was not affected. Bars represent the mean ± the standard error of the mean. Coomassie staining of the blotted membrane shows that equal amounts of protein were loaded into each well of the gel

Calpain activity will augment with increased intracellular calcium; however, we cannot assume that the increased calcium is the only explanation for the increased calpain activity. Thus, we asked if expression and/or protein abundances of either calpains 1, 2, or 3 or calpastatin were affected by HERG1A expression. We used quantitative real-time PCR to discover that HERG1A expression does not produce a statistically significant change in calpain 1 mRNA levels for up to 84 h after viral treatment (Fig. 5a). As well, no change in gene expression was detected for calpains 2 or 3 (data not shown). Further, our results indicate that there is no significant change in calpain 1 protein abundance (Fig. 5b; *n* = 6; Student’s *t* test). Calpain 2, when autolyzed and hence activated, appears as a doublet found at ~ 75 kD [24]. Interestingly, our results show that there is a 40.7% decrease (*p* < 0.05; *n* = 6; Student’s *t* test) in total calpain 2 protein abundance in response to 48 h of HERG1A treatment (Fig. 6). Calpastatin expression was not significantly affected by the HERG1A channel for up to 84 h post-transduction (Fig. 7a); however, calpastatin protein abundance declined by a statistically significant 31.7% (Fig. 7b; *p* < 0.05; *n* = 6; Student’s *t* test). Additionally, there is a decrease in two of the three noted calpain 3 autocatalytic products (25; Fig. 8): the 114 kD isoform is down 29.6% and the 60 kD isoform is down 29.2%, although the 30 kD isoform is not affected (*p* < 0.03; *n* = 6; Student’s *t* test within protein isomer). When the optical densities for all protein bands are summed, there is a total 21.0% decrease in calpain 3 protein abundance.

## Discussion

The ERG1a voltage-gated K^+^ channel is responsible for late phase repolarization of the cardiac action potential and was reported to be absent from skeletal muscle [23, 25]; however, the Pond and Hannon labs demonstrated that this protein is detectable in the atrophying skeletal muscle of mice and in very low abundance in healthy rodent muscle with careful use of protease inhibitors and concentration of solubilized membrane proteins [18]. Subsequent studies showed that ERG1a expression leads to an increase in abundance of the MURF1 E3 ubiquitin ligase protein and enhances ubiquitin proteasome proteolysis, a pathway known to contribute to skeletal muscle atrophy [15, 18]. Here, using C_2_C_12_ myotubes transduced with either control or HERG1A-encoded adenovirus, we show that HERG1A expression also increases basal [Ca^2+^]i and calpain activity. There are numerous potential sources of the calcium that contributes to the increased [Ca^2+^]i. For example, it is possible that ERG1A is modulating Cav1.1 channels in the skeletal muscle sarcolemmal membrane, resulting in an influx potentially from both the external milieu and internal stores. Further, because ERG1A is located in the t-tubules of cardiac tissue [17, 20], it is possible that it is located in the t-tubules of skeletal muscle, where it could contribute to the release of calcium from internal stores by modulation of ryanodine receptors and/or IP3 receptors. Indeed, changes in regulation of sarcolemmal permeability could have severe consequences for skeletal muscle tissue, potentially producing diseases such as muscular dystrophies and Niemann-Pick disease [26, 27]. The source of the increased calcium is currently under investigation in our laboratories. However, because we find no change in the fusion index or an increase in either the number of centrally located nuclei or in the abundance of laminin fluorescence in the fibers over-expressing Merg1a, we believe that our data suggest that the channel (which we find to be in very low abundance in muscle normally) is simply upregulating protein degradation in our myotubes. It is also possible that the low levels of increased calcium are affecting signaling pathways, but that remains to be investigated.

The explanation for the increased calpain activity may seem obvious—the increase in [Ca^2+^]i. However, we ectopically expressed mouse *erg1a* (*Merg1a*) in mouse *Gastrocnemius* muscle and homogenized the muscle, thereby disrupting the [Ca^2+^]i pool and equalizing the calcium concentration throughout the sample. We then assayed for calpain activity and discovered that even in the homogenate it is still higher in the *Merg1a*-expressing tissue. This study is evidence that increased [Ca^2+^]i may not be the only factor that contributes to the ERG1A-induced increase in calpain activity. Other possible contributors include increased calpain 1 and/or 2 protein and/or decreased calpastatin protein.

Calpains 1 (μ-calpain) and 2 (m-calpain) are both classical calpains and are detected throughout the body, including skeletal muscle [28]. Indeed, calpain activity has been demonstrated to contribute to muscle atrophy [28]. For example, Shenkman and colleagues inhibited calpain activity in hind limb suspended mice by treatment with the calpain inhibitor PD150606 and demonstrated that blocking calpain activity reduced the activation of calpain 1 gene expression and attenuated skeletal muscle atrophy [29]. Here, we report that there is no detectable change in calpain 1 protein abundance in myotubes transduced with HERG1A while surprisingly we detect a decrease in calpain 2 protein abundance. These data demonstrate that the increased calpain activity is not a result of increased enzyme protein abundance. We suggest that the decreased calpain 2 protein abundance could result from either decreased calpain 2 synthesis and/or increased calpain 2 protein degradation. Quantitative PCR data demonstrate that there is no significant change in transcription of calpain 1 or 2 genes for up to 84 h post transduction. Interestingly, we observe a decrease in calpain 2 protein abundance without detecting a change in transcription of that gene. Thus, although mRNA production is not always directly correlated with protein abundance, we can speculate that the calpain 2 protein may be undergoing an increased level of degradation. Indeed, these proteins may be undergoing autolysis or it is possible that ubiquitin proteasome proteolysis of calpain 2 is enhanced. Indeed, we have shown that increased ERG1 expression increases UPP activity.

Calpastatin is a native calpain inhibitor which inhibits conventional calpains 1 and 2, but not calpain 3. Calpastatin requires calcium to bind calpains so that when the calcium concentrations rise, calpain activity is increased, but so is calpastatin binding [13, 30]. Indeed, a decrease in calpastatin protein would lower the inhibition of calpains and allow for increased calpain-mediated proteolysis. Certainly, the increased level of calpain activity assayed in the mouse muscle homogenates, in which the [Ca^2+]^i is disrupted, suggests that something other than [Ca^2+^]i must contribute to enhanced calpain activity.

Calpain 3 is a non-classical calpain which is detected mainly in skeletal muscle. It undergoes calcium-mediated autolysis that has been reported to be enhanced by ATP at lower calcium concentrations [31, 32]. Evidence has shown that the absence of calpain 3 leads to a reduction in protein turnover and results in accumulation of damaged and/or misfolded proteins which can lead to cellular stress and eventual muscle pathology [33, 34]. Indeed, the absence or reduction of this protein has been shown to lead to limb-girdle muscular dystrophy type 2A (LGMD2A) in humans [30–32, 34–37]. Studies suggest that calpain 3 takes part in remodeling of the sarcomere in response to cellular damage such as atrophy [34, 36, 37]. Interestingly, studies with calpain 3 knockout mice suggest that calpain 3 acts upstream of the UPP, although it is uncertain if calpain 3 directly cleaves proteins to make them accessible for ubiquitination [34]. Thus, calpain 3 appears to be protective against muscle loss and its protein abundance might be expected to be lower in an atrophic situation. Indeed, we report that calpain 3 protein abundance decreases in response to HERG1A expression. The decrease may be related to a decreased ability to remodel the sarcomere during/after atrophy; however, this possibility would require much additional investigation.

In summary, we show that HERG1A increases calpain activity in myotubes, likely resulting from the increase in [Ca^2+^]i. We detect no increases in abundances of calpains 1 or 2 proteins which would otherwise contribute to enhanced calpain activity. In fact, we report a decline in the abundance of calpain 2 protein. Thus, it would appear that the increased [Ca^2+^]i could be the main contributor to the enhanced calpain activity; however, there is a significant decline in calpastatin protein abundance which likely also contributes to the measured increase in calpain activity. This is not surprising considering that calpastatin binding is also enhanced by intracellular calcium. Calpain 3 activity was not measured here; however, the decline in calpain 3 protein is consistent with an atrophic environment. Interestingly, classical calpain activity has been shown to degrade sarcomeric anchor proteins (e.g., titin, nebulin) and this allows for release of contractile proteins (e.g., myosin and actin) into the cytosol where they can be accessed and degraded by the UPP [30, 38]. Here, we show that HERG1A modulates intracellular calcium and calpain activity. Because its interaction with calcium and calpains is upstream of the UPP, and it also modulates UPP activity [18], we hypothesize that ERG1A may indeed contribute to coordination of proteolytic systems which produce skeletal muscle atrophy, specifically calpain and UPP activities. Further study is needed to learn how ERG1A functions in skeletal muscle. Indeed, because of the role of the ERG1A/ERG1B heteromultimeric channel in cardiac action potential repolarization, ERG1A will likely never be a target for pharmacological treatment of atrophy; however, continuing study of this protein may reveal other possible targets to combat atrophy.

## Data Availability

The datasets used and/or analyzed during the current study are available from the corresponding author on reasonable request.

## Abbreviations

DMEM: Dulbecco’s modification of Eagle’s medium
*ERG1A*: *Ether*-*a*-*gogo*-*related gene*
FBS: Fetal bovine serum
*HERG1A*: Human *ether*-*a*-*gogo*-*related gene*
*Merg1a*: Mouse *ether*-*a*-*gogo*-*related gene*
RLU: Relative light units
UPP: Ubiquitin proteasome pathway
